# Method for the Development of WISH, a Globally Applicable Index for Healthy Diets from Sustainable Food Systems

**DOI:** 10.3390/nu13010093

**Published:** 2020-12-30

**Authors:** Laura Trijsburg, Elise F. Talsma, Sandra P. Crispim, James Garrett, Gina Kennedy, Jeanne H. M. de Vries, Inge D. Brouwer

**Affiliations:** 1 Human Nutrition and Health, Wageningen University and Research, 6700 AA Wageningen, The Netherlands; elise.talsma@wur.nl (E.F.T.); jeanne.devries@wur.nl (J.H.M.d.V.); inge.brouwer@wur.nl (I.D.B.); 2 Department of Nutrition, Federal University of Paraná, Curitiba 80210-170, Brazil; crispim@ufpr.br; 3 Bioversity International, 00054 Rome, Italy; J.Garrett@cgiar.org; 4 Advancing Nutrition, USAID, Arlington, VA 22202, USA; gina_kennedy@jsi.com

**Keywords:** diet quality, sustainable diet, index, food system

## Abstract

Promoting both a healthy diet and at the same time considering the environmental sustainability aspects of production and consumption of the diet are urgent global issues. We developed the WISH (World Index for Sustainability and Health) to evaluate diets for healthiness and sustainability. The WISH seeks to measure two complex multidimensional concepts, diet quality and environmental sustainability, in one scoring system. The WISH is based on the EAT-Lancet recommendations for a healthy and sustainable diet in the general population with global applicability across multiple settings. Thirteen food groups are scored between 0 and 10, based on their association with disease and impact on environmental indicators. The scoring system was applied using a dataset of duplicate 24 h dietary recalls from 396 urban Vietnamese men and women. Out of a maximum score of 130, the mean total WISH score was 46 (SD 11), and scores for the healthy and high-environmental impact sub-scores were mean 25 (11) (out of 100) and mean 26 (8) (out of 70) respectively. A higher score was observed for the less-healthy (mean 20 (2) out of 30) sub-score. Our initial analysis shows that the WISH is able to differentiate between the healthiness and the environmental sustainability of a Vietnamese diet.

## 1. Introduction

Current food systems are being increasingly challenged to provide healthy food for all, while doing so in a sustainable and equitable way. Worldwide, diets of poor quality are the main contributors to morbidity and mortality as well as all forms of malnutrition [1,2,3], exceeding the burdens attributable to many other global health challenges [1]. While some progress has been made on decreasing the prevalence of undernutrition (stunting and wasting), micronutrient deficiencies persist and the prevalence of overweight, obesity and diet-related non-communicable diseases are rising across the globe, the fastest in low-income countries [3,4]. Promoting healthy diets is one of the major strategies to prevent both undernutrition and micronutrient deficiencies, and mitigate the rise of overnutrition and diet related non-communicable diseases (NCD) [3,5,6]. A healthy diet is comprised of an intake of adequate amounts of fruits, vegetables, whole grains, legumes and nuts; sufficient intake of animal source foods (milk, egg, poultry, fish in particular), further referred to as protective foods; and an avoidant or limited intake of foods, food groups, and nutrients that form health risk factors when eaten in excess, including free sugars (including sugar sweetened beverages), saturated and trans-fat, salt, red meat, processed meat, and ultra-processed foods [1,7], further referred to as limiting foods.

Adding considerations beyond health creates a complex problem. There is growing pressure for agricultural production to intensify to meet rising global demands for animal source foods, fish, fruits and vegetables, as well as cereals and legumes. Food is being transported farther from where it is grown to where it is eaten, creating mixed consequences for diets [8]: increasing the environmental pressure [9] as well as risks of food safety [10], depending on the context. Various indicators for measuring the impact of a certain food (group) on the environment exist, where greenhouse gas emission (GHGe) is the most often used in research. Other indicators are, e.g., blue water use, land use, biodiversity, eutrophication or pesticide use. Generally, animal source foods have a higher environmental impact than plant based foods (regardless of the environmental indicator used) [11,12,13,14], where especially ruminant meat (such as beef, goat and sheep) shows a high impact on the environment irrespective of the environmental indicator [12]. Plant-based food groups with a relatively low impact on the environment include fruits, vegetables, cereals, potatoes and legumes and sugar-sweetened beverages [13,14]. It should be mentioned that within food groups a large variety exists in environmental impact of the food items, depending on the systems of production, transportation, and transformation used (e.g., whether fish is wild caught or from trawling fisheries, the amount of forest clearing undertaken, or complexity and length of the supply chain), or whether the fruits are in season imported fruits or fruits from greenhouses pose a larger pressure on the environment) [13,15].

Characteristics of diets with low environmental impact are broadly similar to those of a healthy diet across multiple settings [11,15]. Animal source foods (ASFs) are among the most contested elements of these diets because their production generally has higher negative environmental impacts and they are consumed in quantities higher than healthy diet recommendations. However, ASFs are some of the best dietary sources of essential nutrients such as iron, calcium and B vitamins. In particular, vitamin B12 is obtained from ASFs unless fortified foods or supplements are available, which is not the case in many countries. Therefore, worldwide, but especially in lower and middle income countries (LMICs), ASFs are considered an essential part of the diet, especially for infants and young children [16].

To assess whether a population consumes a healthy diet, numerous diet quality indices are available, though the majority of these are developed for a specific country or region and such indices are often designed for use in high-income countries/regions [17]. The outcomes of indicators assessing the impact of the diet on the environment can vary tremendously across countries for the same food product as sustainability depends on the way the product is produced, stored, transported, processed and packaged. Indices combining the healthiness and environmental impact of the diet in one score are available [18,19]. However, such indices have been developed for high-income countries and need information from life-cycle assessment databases, which are often not available for many products consumed in LMICs [20]. Therefore, there is a need for a relatively simple index using minimal data that measures both healthiness and environmental impact of the diet.

The challenge is to find an optimum balance where a healthy diet goes hand in hand with a sustainable diet, without compromising too much on the quality of the diet on the one hand, or the sustainability of the food system that delivers the diet on the other. This paper describes the development of the WISH (World Index for Sustainability and Health), an index that is based on globally applicable recommendations for a healthy diet for the general healthy adult population within environmental sustainability targets. Optimal levels of intake of different food groups to promote health are formulated by WHO [7] or the global burden of disease (GBD) study [1], but do not take environmental sustainability into account. Although it has suffered some criticisms [21], the EAT-Lancet recommendations are the first attempt to develop a global set of dietary guidance where sustainability is taken into account alongside healthiness of the diet. We therefore used this guidance to develop the WISH [22].

## 2. Materials and Methods

### 2.1. Development of the WISH

The WISH aims to score the healthiness and the environmental impact of the diet in one scoring system. Scoring of the WISH does not require food composition table (FCT) or life-cycle assessment data (LCA) as these are not available for all countries or regions. The WISH is designed to monitor the healthiness and environmental sustainability of the diet of a population at a certain timepoint and can also be used to monitor changes over time and allows for comparison amongst different population groups in different countries or regions.

#### 2.1.1. Components of the WISH

The individual components of the WISH are based on the set of recommendations for food groups from the EAT-Lancet report [23]. The EAT-Lancet report includes recommendations for 14 foods and food groups: whole grains, tubers and starchy vegetables, vegetables, fruits, dairy foods, red meat, fish, eggs, chicken and other poultry, legumes, nuts, unsaturated oils, saturated oils, and added sugars. Although we recognize that tubers and starchy vegetables are important components of the diets of many populations, we excluded them from this analysis, as since the amounts set in the EAT-Lancet diet for whole grains and roots and tubers were also increased to ensure the energy intake of the proposed diet reached 2500 kcal/day, thus the recommended amounts are not solely based on disease prevention or environmental impact. Moreover, neither WHO nor GBD recommendations include tubers and starchy vegetables. Hence, the index described in this paper consists of 13 foods and food groups (see Table 1). The EAT-Lancet recommendations for classifying a food group as protective, neutral, or limit are based on existing systematic reviews, meta-analyses and pooled analyses of primary data (see Table 1 for the classification for each food group). The EAT-Lancet commission did not perform a new systematic review, but based their recommendations on a selection of the review work done by others. The list of studies can be found in the supplementary material accompanying the EAT-Lancet recommendations [22].

Environmental impact is based on the findings of Clark et al. [13] and includes five sustainability indicators: greenhouse gas (GHG), land use, eutrophication, acidification, and scarcity weighted water [13]. The indicated environmental impact in the WISH index is not specified for the individual foods but for the food groups as a whole. For a detailed description of estimating the environmental impact for a food group as a whole we refer to the supplementary material of Clark et al. [13]. Clark et al. classifies food groups as low, medium or high environmental impact. We use this as a generic summary of the impact of a food group on the environment for construction of the WISH. Individual food items in a food group can have a very different impact on the environment thus context in life cycle assessment (LCA) is important. See Table 1 for the environmental impact classification of each food group.

#### 2.1.2. Scoring of the WISH

All 13 components are scored between 0 and 10, where 0 indicates no adherence to our set of reference recommendations and 10 indicates complete adherence. Higher scores on the individual components and the total and sub-scores indicate a healthier, environmentally friendlier diet. Scores are assigned within the range of 0 to 10 assuming a linear relationship between the component and the health outcomes [24].

For all the protective food groups, a certain amount of the food group needs to be consumed before the full health benefits are observed. Assuming a linear relationship between the component and the health outcomes, for a consumption between the lower recommended intake and the recommended intake, a score between 0 and 10 is assigned according to the following formula:

(1) $$10\times \;\frac{reported\;intake-lower\;recommended\;intake}{recommended\;intake-lower\;recommended\;intake}$$

For the food groups, whole grains, fruits, vegetables and legumes, no restrictions at the upper ranges are modeled. Consuming the recommended, upper or an intake above the upper recommended intake level results in the maximum score of 10.

For the food group unsaturated oils consumption between the recommended intake level and the upper recommended intake level is scored as 10. However, as a higher intake increases the energy consumption and could thus contribute to overweight and obesity, the score is capped at the upper recommended intake. This means that an intake above the upper recommended intake is scored as 0.

For the protective food groups nuts, dairy foods and fish intakes between the recommended intake and the upper recommended intake are scored as 10. However, as these food groups pose a medium or high impact on the environment, intakes above the upper recommended intake are assigned a score of 0 given the impact on the environment.

For red meat, saturated oils and added sugars consumption should not exceed the recommended amount, as negative health effects increase from that intake level onwards. For the two neutral healthy groups (eggs, and chicken and other poultry) a medium environmental impact is observed, with higher intakes consequently increasing the burden on the environment. Therefore, an intake between the lower recommended intake (set at 0 g for all 3 food groups) and the recommended intake by EAT-Lancet is scored as 10. An intake between the recommended amount and the upper recommended intake is scored between 10 and 0 assuming a linear relationship and an intake exceeding the upper recommended intake is scored as 0. The score for the intake between the recommended intake and the upper recommended intake is calculated according to the formula below:

(2) $$10\times \;\frac{\left(upper\;recommended\;intake-recommended\;intake\right)-\;\left(reported\;intake-recommended\;intake\right)}{upper\;recommended\;intake-recommended\;intake}$$

For saturated oils and added sugars, no upper range of intake level is given by the EAT-Lancet recommendations. Both components are scored as a bi-variate component (so a score of either 0 or 10 is obtained). A consumption between 0 and the recommended intake was scored as 10 and intakes above this recommended level are scored as 0 points.

For the calculation of the total WISH score, all components are summed up and are given equal weight in the total score. However, when summing up everything in one score a diluting effect is expected [25]. Consuming a significant amount of the more desirable food groups along with a significant amount of less desirable ones can give the same score as if an individual consuming a moderate amount of each. A differentiation between these two cases cannot be made based on the total score, but a healthy and less healthy score can show the difference in dietary pattern between the two. The same holds for a mixed diet with high and low environmental impact foods. Thus to overcome this diluting effect four sub-scores are proposed. Two are based on the diet quality concept (the healthy and the less healthy sub-scores) and two are based on the environmental sustainability of the food group (the high and low environmental impact sub-scores). The sub-scores give a more refined view on what problems exist in a certain region or country and what researchers and policymakers should focus on to lift the dietary intake of the region or country to a healthier and more sustainable dietary pattern.

- *Healthy sub-score*: Summing the 8 protective and 2 neutral food groups: whole grains, vegetables, fruits, dairy, fish, eggs, chicken and other poultry, legumes, nuts and unsaturated oils. A higher sub-score for protective foods means a higher adherence to the recommendations for these protective foods and thus a healthier diet.
- *Less healthy sub-score*: Summing the 3 limiting food groups: red meat, saturated oils and added sugars. A higher sub-score for limiting foods means a higher adherence to the recommendations for these limiting foods and thus a healthier diet.
- *Low environmental impact sub-score:* Summing the 6 low environmental impact food groups: whole grains, vegetables, fruits, legumes, unsaturated oils, and added sugars. A higher sub-score for low environmental impact foods means a higher adherence to the recommendations for these low environmental impact foods and thus a relatively low impact on the environment. A low sub-score means primarily that consumption is below recommended amounts for the components (whole grains, vegetables, fruits, legumes), since more of these foods could be consumed as part of a healthy diet, with relatively limited impacts on the environment. However, for the unsaturated oils and added sugars food groups consumption should not exceed the recommendations due to possible health constrains.
- *High environmental impact sub-score:* Summing the 4 medium and 3 high environmental impact food groups: saturated oils, dairy foods, red meat, fish, eggs, chicken and other poultry and nuts. A higher sub-score for high environmental impact foods means a higher adherence to the recommendations for these high environmental impact foods and thus a relatively low impact on the environment. A lower sub-score would mean consumption beyond recommended amounts and so greater negative impacts on the environment.

Below is a detailed description of the 13 food groups that are included in the WISH with reference to their association with health, their impact on the environment, a listing of the food items in each group, and description of their scoring. No information from an FCT is needed for the scoring of the different components.

##### Whole Grains

This is a protective food group with relatively low impact on the environment [15]. Health benefits of whole grains were observed on total mortality, cardiovascular disease (CVD), and cancer [22]. Generally whole grain cereals have a low environmental impact (on land use, eutrophication, acidification, and scarcity weighted water), although the production of rice is high in GHG emissions [13]. The intake of this food group can be defined as the average daily consumption of whole grains such as rice, wheat and corn (bran, germ, and endosperm in their natural proportion) or whole grain products in the form of breakfast cereals, bread, pasta, biscuits, muffins, tortillas, pancakes, and other sources. The amount recommended by the EAT-Lancet commission for this food group originates from its benefits for health, but the recommended intake of this food group was increased to achieve the average requirement of 2500 kcal/day of the recommended diet. Thus, the recommendation most likely exceeds the minimum amount necessary for a healthy diet, and therefore the recommended amount of the Global Burden of Disease study [1] was used for this food group. This recommendation is an intake of 125 g/day or higher of whole grains, and this was scored as 10. The recommended range of intake starts at 100 g/day. Thus, an intake below 100 g was scored as 0. An intake between 100 and 125 g/day was proportionally scored between 0 and 10.

##### Vegetables

This is a protective food group with relatively low impact on the environment. No distinction between different types of vegetables (dark green, red and orange or other vegetables) was included here as it is not clear to the authors how the EAT-Lancet arrived at the amounts for the different vegetable groups. Health benefits of vegetable consumption are seen for mortality, cancer and CVD [22]. Environmental impact of vegetables can be low, although higher GHG emissions are observed for vegetables grown in heated greenhouses whereas land use decreases for such vegetables compared to vegetables grown in open fields [13]. This food group is defined by EAT-Lancet as fresh, frozen, cooked, canned, or dried vegetables, and excludes legumes and salted or pickled vegetables, juices, nuts, and seeds, and starchy vegetables such as potatoes or corn. The recommendation is an intake of 300 g/day or higher of vegetables, which is scored as 10. The recommended range of intake starts at 200 g/day. Thus, an intake below 200 g was scored as 0. An intake between 200 and 300 g/day was proportionally scored between 0 and 10.

##### Fruits

This is a protective food group with relatively low impact on the environment. The EAT-Lancet commission does not make a distinction in different fruits such as vitamin A rich or citrus fruits. Health benefits of fruit consumption are seen for mortality, cancer and CVD [22] and environmental impact of fruits are generally low, although like for vegetables, higher GHG emissions are observed when fruits are grown in heated greenhouses, whereas land use decreases compared to when fruits are produced in open fields [13]. The food group is defined as fruits, which are fresh, frozen, cooked, canned, or dried but excluding fruit juices, salted or pickled fruits and nuts or seeds. The recommendation is an intake of 200 g/day or higher of fruits and is scored as 10. The recommended range of intake starts at 100 g/day. Thus, an intake below 100 g was scored as 0. An intake between 100 and 200 g/day was proportionally scored between 0 and 10.

##### Dairy Foods

This is a protective food group with medium impact on the environment. Health benefits of dairy consumption are observed on cancer occurrence [22] with medium impact on the environmental indicators, although with considerable variation of indicator values depending on geographical location and production system [13]. The definition of dairy foods includes whole or skimmed milk or derivative equivalents (e.g., cheese, yoghurt or curd), excluding butter and cream. As some form of diary is needed in the diet to provide calcium intake, the optimum range of intake of 250–500 g/day was scored at 10 points. An intake between 0 and 250 g/day was scored as a ratio between 0 and 10, whereas an intake higher than 500 was scored as 0 given the increased pressure such intakes pose on the environment.

##### Red Meat

This food group is considered a limiting food group with relatively high impact on the environment. No distinction between red meat from ruminants or non-ruminants is made in the EAT-Lancet classification. Red meat is associated with negative health impacts on mortality, cancer, type 2 diabetes and CVD [22]. Furthermore red meat has the highest environmental impact in all five environmental indicators [13]. Red meat is beef, pork, lamb, and goat including red meat in its processed form but excluding poultry, fish, eggs. The maximum intake without negative health implications was set at 14 g/day or less while the upper range value of 28 g/day was set to score 0. A consumption between 14 and 28 g/day was scored as a ratio between 0 and 10.

##### Fish

This is a protective food group with relatively high impact on the environment. Fish consumption is associated with health benefits for CVD and total mortality [22]. Fish has a high impact on the environment, although this depends on the type of fish and the production method [13]. This food group includes fish and shellfish such as mussels and shrimp but no distinction for fish rich in omega-3 was made. As some amount of fish is needed in the diet to provide its health benefits the optimum range of intake of 28–100 g/day was scored at 10 points. An intake between 0 and 28 g/day was scored as a ratio between 0 and 10, whereas an intake higher than 100 was scored as 0 given the increased pressure such intakes pose on the environment.

##### Eggs

This is a neutral food group with medium impact on the environment. Egg consumption is not specifically linked to either beneficial or detrimental health effects for the adult population [22]. Sustainability indicators show a medium environmental impact [13]. This food group includes eggs from chickens and ducks, but excludes fish eggs. The maximum score of 10 is obtained if 13 g/day or less is consumed, based on the impact on the environment. The upper range value of 25 g/day was set to score 0. A consumption between 13 and 25 g/day was scored as a ratio between 0 and 10.

##### Chicken and Other Poultry

This is a neutral food group with medium impact on the environment. Chicken and poultry consumption is not specifically linked to either beneficial or detrimental health effects [22], whereas the indicators show a medium environmental impact given the amount of feed needed for meat production [13]. This food group includes meat from chicken, ducks, geese etc. The maximum score of 10 is obtained if 29 g/day or less is consumed, while the upper range value of 58 g/day was set to score 0. A consumption between 29 and 58 g/day was scored as a ratio between 0 and 10.

##### Legumes

This is a protective food group with relatively low impact on the environment. Legumes have been associated with health benefits for CVD and mortality [22] whereas the environmental impact of this food group is low, partly due to legumes’ ability to catch nitrogen from the atmosphere and store it in their root system [13]. This food group includes fresh, frozen, cooked, canned, or dried legumes, beans and lentils, but also includes soy foods and peas. An intake between 0 (lower recommended range of intake) and 75 g/day was scored as a ratio between 0 and 10, while an intake of 75 or higher was scored as 10.

##### Nuts

This is a protective food group with intermediate impact on the environment. Health associations between nuts and type II diabetes and mortality have been observed [22]. The environmental impact is low, although the scarcity weight water use for different types of nuts is highly variable [13]. This food group includes tree nuts and ground nuts (including peanuts). The optimum range of intake of 50–75 g/day was scored at 10 points. An intake between 0 and 50 g/day was scored as a ratio between 0 and 10, whereas an intake higher than 75 was scored as 0 given the increased pressure such intakes pose on the environment.

##### Unsaturated Oils

This is, within the indicated range, a protective food group with relatively low impact on the environment. Positive health impacts of unsaturated oils are observed for mortality and CVD [22], and environmental impact is low [13]. Examples of unsaturated oils are olive, soybean, rapeseed, sunflower and peanut oil. The recommendation is an intake of 40 g/day or higher and is scored as 10. The recommended range of intake starts at 20 g/day. Thus, an intake below 20 g was scored as 0. An intake between 20 and 40 g/day was scored proportionally between 0 and 10, whereas an intake higher than 80 was scored as 0 given that a high intake of fats increases the energy intake and thus contributes to overweight and obesity.

##### Saturated Oils

This is a limiting food group with relatively high impact on the environment, and scored as a bi-variate component (so a score of either 0 or 10 is obtained). Health impact for saturated oils intake are seen for CVD [22] and the environmental impact is high, given the burden posed on the environment by palm oil [13], and animal-based fats have a higher impact on the environment than the plant-based oils. Saturated oils originate from oils like palm oil, and from dairy fats, lard or tallows. Only food item information is needed for the scoring. The cut-off value for scoring 10 points was set to an intake of 11.8 g/day, intakes above this recommended level are scored as 0 points.

##### Added Sugars

This is a limiting food group with relatively low impact on the environment, and scored as a bi-variate component (so a score of either 0 or 10 is obtained). Negative health impact of the consumption of sugars is seen for type II diabetes and CVD [22], whereas the environmental impact is amongst the lowest of the food groups of this index [13]. Added sugars include measured added sugar and sugar sweetened beverages. The cut-off value for scoring 10 points was set to an intake of 31 g/day, intakes above this recommended level are scored as 0 points.

### 2.2. Example Calculation of the WISH

The above described WISH was applied using dietary intake data from a Vietnamese population. In this population the total WISH score as well as sub-scores and individual component scores were calculated.

#### 2.2.1. Dataset

The dataset on dietary intake collected in the framework of the BMGF/FCDO funded project on Fruits and Vegetable Intakes in Vietnam and Nigeria was used (https://anh-academy.org/increasing-fruit-and-vegetable-intake-low-income-populations-vietnam-and-nigeria-through-food). The target population was composed of males and non-pregnant females, 18–49 years old from low-income households, 200 from a peri-urban and 200 from an urban area of Hanoi in Vietnam. Data were collected between March and April 2019. Low income was defined as a monthly household income below 2.65 million Vietnamese Dong (VND), per capita within the household. This income criterion was used to create a list of eligible households by the responsible government bodies resulting in 458 and 639 eligible households for the urban and peri-urban area respectively. Households were selected randomly in each area from this household list. All subjects gave their informed consent for inclusion before they participated in the study. The study was conducted in accordance with the Declaration of Helsinki, and the protocol was approved by the Hanoi Medical University ethical committee (approval number 45–18/HMUIRB).

#### 2.2.2. Dietary Intake Data Collection

Dietary intake of adults (both women and men from the same household) was assessed by using a multi-pass quantitative 24 h recall (24hR) method. Duplicate, non-consecutive 24hRs were conducted for all, carried out with a difference of at least two days between the recalls.

Conversion factors for the cooking method, waste factors, total volume cooked, and portion consumed were used to calculate the amount of food item consumed in grams. The individual food items were grouped into the WISH food groups based on the description given by the EAT-Lancet commission [22,22]. The dietary data were entered in Compl-eatTM (Wageningen University, Wageningen, The Netherlands, Version 1.0). Recipes were disaggregated into ingredients to classify them into the food groups. Data were cleaned by calculating z-scores. Recalls with energy intake outside +2.58 or −2.58 z-scores were checked for mistakes with the original paper questionnaires and corrected where possible.

#### 2.2.3. Statistical Analysis

Analysis was performed using R version 3.6.1. All food intakes were averaged over the two collection days before being used to score individual dietary intakes. To provide insights into the relation amongst the components and to determine which components have the highest influence on the total and sub-scores of the WISH, Spearman’s correlations were calculated where all food groups were included in the total score and correlated to the individual scores. Only dietary intake data of persons with two 24-h dietary recalls were maintained (*n* = 396).

## 3. Results

**Figure 1:**
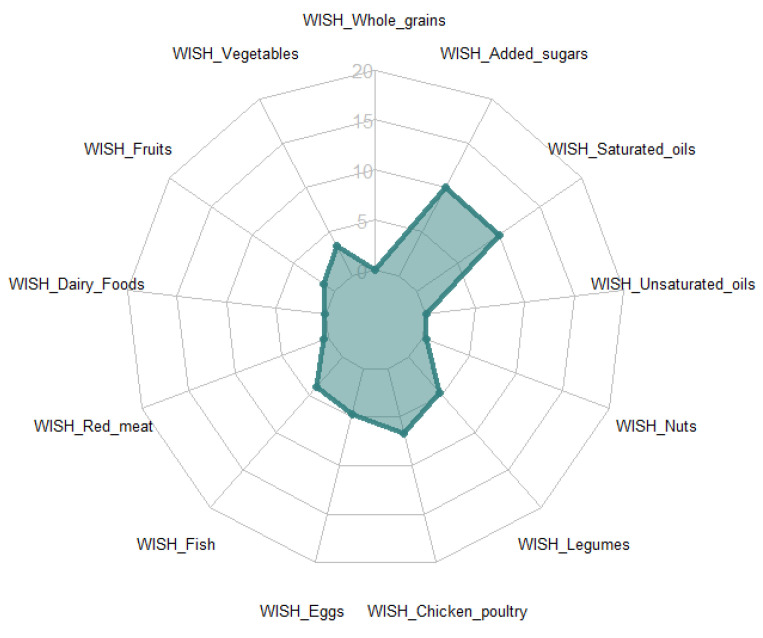
Mean scores of the components of the WISH in a Vietnamese population.

The diet of this Vietnamese population is predominantly comprised of white rice, combined with vegetables and red meat. Fish, eggs, chicken and legumes are also consumed but by a smaller part of the population. A higher score indicates a healthier and more sustainable dietary pattern, both for the component scores and for the total and sub-scores. Figure 1 shows the mean scores of the 13 component scores. This Vietnamese population scored high on 2 of the limiting diet component scores, namely saturated oils (mean 10 (SD0.7) out of 10) and added sugars (10 (0.7)).

Low intakes and consumption of these food groups (saturated oils mean intake 0.2 g with 90.7% non-consumers and for added sugars a mean intake of 1.1 g with 81.1% non-consumers, Table 2) were observed, meaning that the intakes were far below the recommended dietary intakes by the EAT-Lancet commission and so, in actuality, healthier. Low scores were observed for whole grains (mean 0), fruits (mean 1.3), dairy foods (mean 0.1), nuts (mean 0.4) and unsaturated oils (mean 0.1) due to the high percentage of non-consumers, and an intake below the recommended intake for fruits, dairy foods, nuts and unsaturated oils. The recommendation for these food groups would be to increase their intake to obtain higher scores. Scores for red meat (mean 0.5) and fish (mean 3.9) were also low as intakes often exceeded the EAT-Lancet recommendations. To increase WISH scores for these food groups, intakes should decrease. In this sample, 39.6% and 55.1% did not consume eggs and chicken and poultry respectively. This part of the population could increase their intake to a maximum of 15 g for eggs and 30 g for chicken and poultry while still scoring high on the WISH. Though the part of the population that does consume these food groups consumes above the recommended intake 25.1 g for eggs and 37.7 g for chicken and poultry, for that group it is recommended to lower their intake.

**Figure 2:**
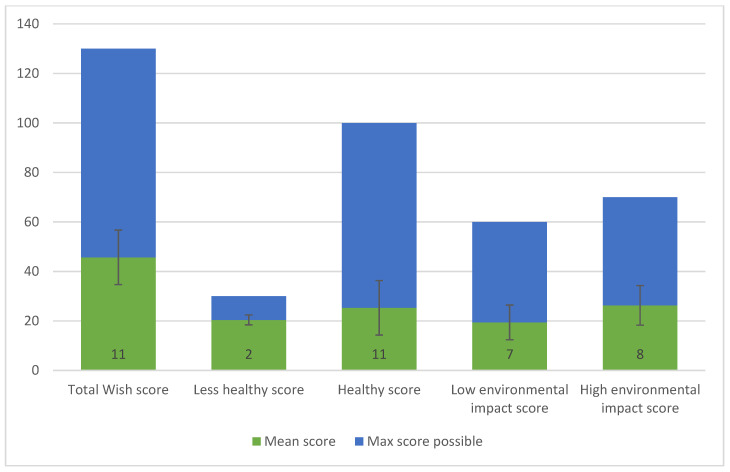
Total and sub-scores for the WISH in a Vietnamese population SDs are given below the error bars.

Higher mean scores on the total and sub-scores indicate the population consumes a healthier and more sustainable diet. This also holds for the less healthy and low environmental impact sub-scores, as the component scores are inversely scored a higher mean score on the less healthy sub score indicates a consumption within the recommended intake range of the moderation components. A higher mean score on the low environmental impact sub score indicates the components with a medium or high environmental impact are consumed within the recommended range of intake and are thus within the planetary boundaries. The total mean WISH score in this Vietnamese population was 45.7 (SD 11) out of 130 (Figure 2). This population scored relatively high on the less healthy sub score (20.4 out of 30), due to the high mean component scores of 2 (saturated oils and added sugars) out of 3 components included in this sub-score. However, a low score was observed for the healthy sub-score (25.3 out of 100) due to the low scores on whole grains, vegetables, fruits, dairy foods, and nuts (because of mean consumption of the population below the recommended intake) and low scores on fish and unsaturated oils because of higher consumption than recommended. The high environmental impact sub-score scored 26.3 (out of 70) due to the low component scores of dairy foods, nuts (consumed less than recommended) and red meat and fish (because of higher consumption than recommended).

**Figure 3:**
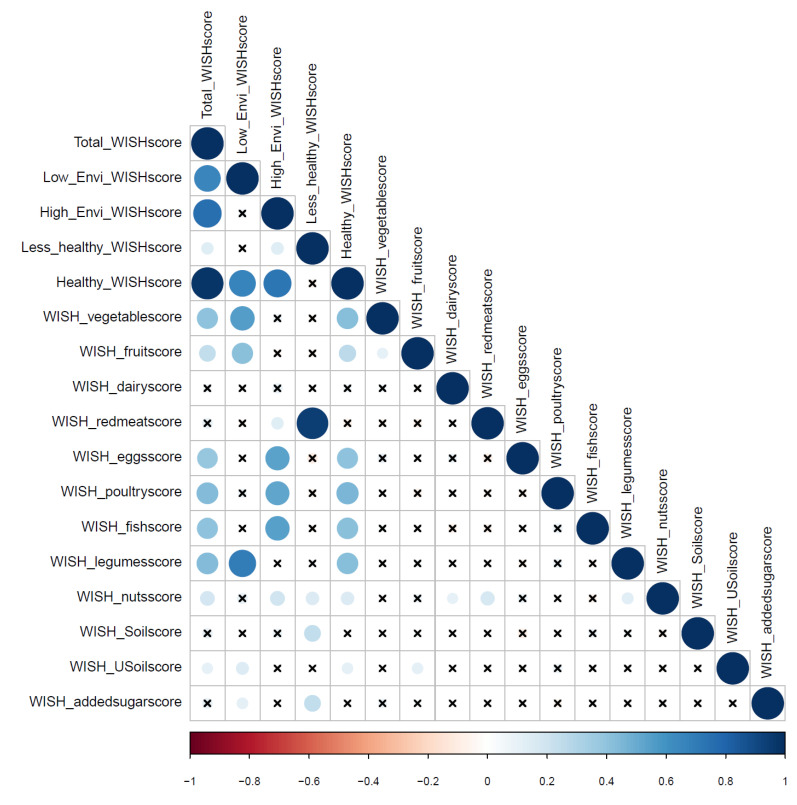
Heatmap of Spearman correlations amongst components scores, total WISH and sub scores (insignificant *p* values (significance level *p* ≤ 0.05) are crossed), the WISH score for whole grains is not shown as the majority of the population scores 0, and thus no correlation could be made. Larger circles mean a stronger correlation (for positive and negative correlations). WISH_USoilscore: unsaturated oil WISH score; WISH_Soilscore: saturated oil WISH score.

Figure 3 shows the heatmap of the Spearman’s correlations amongst the component scores, total WISH score, and sub-scores. The color (red for negative and blue for positive) and size (smaller size means a lower correlation coefficient) of the dot indicate the direction and strength of the Spearman’s correlation coefficient. The total WISH score (including all individual scores) in the Vietnamese population is mainly influenced by the following component scores: legumes (r = 0.43), poultry (r = 0.43), eggs (r = 0.39), fish (r = 0.40), fruits (r = 0.25) and vegetables (r = 0.41) (see Figure 3). Regarding the sub-scores the red meat component has the highest influence on the less healthy WISH sub-score (r = 0.95), whereas the healthy WISH sub-score is influenced by the same component scores as the total WISH score. The low environmental impact WISH sub-score was mainly influenced by the components scores of legumes (r = 0.70), fruits (r = 0.41) and vegetables (r = 0.55) and the high environmental impact WISH sub-score by the component scores of poultry (r = 0.53), egg (r = 0.54) and fish (r = 0.55).

## 4. Discussion

This article describes the development process of the WISH and its application using dietary intake data from a Vietnamese population. Based on the WISH, the diet of this Vietnamese population can be characterized as having an insufficient intake of healthy foods and intake of foods with a high environmental impact could also be reduced. WISH scoring of this population could be improved by increased intake of whole grains, vegetables and fruits. The consumption of dairy foods and nuts can be slightly increased, but with moderation to stay within the bandwidth of EAT-Lancet recommendations. Chicken and poultry and egg consumption should be decreased for the consumers, though the non-consumers could still increase their intake but with moderation. Red meat and fish consumption should be lowered and one could think of substituting this with dairy, chicken and poultry, eggs and nuts. The limiting food groups added sugars and saturated oil consumption are within the recommended range, and consumption levels for these food groups should be maintained or reduced.

Overall, it can be concluded that a major dietary shift is needed in this Vietnamese population to meet the EAT-Lancet recommendations. However, our study population are low income groups living in Hanoi, so the results cannot be generalized to the entire Vietnamese population. It should also be noted that the relatively low WISH sub-component scores could be due to the fact that the participants were selected from low-income households. Vietnam, being a middle-income country, has seen a steep increase in ASF consumption in the past 35 years [26]. Though, calcium deficiency, which is linked to dairy intake, is high amongst the Vietnamese population, with 83% of the women suffering from mild hypocalcemia in 2009 [26,27]. ASFs pose a larger burden on the environment compared to plant-based foods; however, a certain amount of ASF is needed to meet nutrient recommendations [16]. This trade off, which can be expressed as eating ASFs where nutritionally necessary but trying to keep the impact on the environment as low as possible, can thus mean the intake of one of the ASF food groups should be increased (in this case dairy, having a medium environmental impact) while intake of another ASF food group should be reduced (red meat, as it has a high environmental impact), but this all depends on the production systems used and thus needs contextualization.

The EAT-Lancet recommendations recognize that major dietary changes are needed to address the negative influence of the diet on health and environmental sustainability. Another approach, which is used for many national food based dietary guidelines (FBDG) often take into account the current dietary pattern and recognize that even small deviations towards a healthy dietary pattern can lead to positive health outcomes [28,29]. It is thus not surprising that a modeling study showed that adhering to the EAT-Lancet recommendations showed a greater reduction in mortality compared to many national FBDG [30]. Though, another study found that changing energy consumption to prevent underweight, overweight and obesity alone accomplishes a reduction in mortality in the US similar to the mortality reducing effect of the EAT-Lancet diet [21]. Regarding morbidity reduction, adherence to the EAT-Lancet diet showed lower risks of ischemic heart disease and diabetes in a UK study [31]. The majority of the available literature on diet-related diseases, also those used as evidence to set the EAT-Lancet recommendations, come from high-income countries. Although it is assumed that this also gives an indication of leading dietary risk factors in LMIC, diet disease risk patterns might differ for the different regions and more evidence should be generated on this topic.

Following a dietary pattern as recommended by the EAT-Lancet commission was shown to minimize the impact on the environment compared to currently consumed diets in many countries [30]. Underlying this modeling study are Life Cycle Assessment (LCA) data. Such LCA data for LMIC are based on high income countries hence not context specific. Knowledge gaps for LCA data for LMICs should be addressed and future research should focus on local and regional information, as the available evidence should be further contextualized to identify priority food system changes to improve environmental sustainability in LMICs.

The WISH is developed taking into account ease of use while at the same time trying to combine two dimensions, the healthiness and environmental impact of the diet, in one scoring system. These two concepts are modeled in one scoring system using cut-off points, based on recommendations of the EAT-Lancet commission for the 13 food groups. This scoring system was adopted, as the EAT-Lancet commission considered environmental factors and health factors when setting the recommended consumption amounts for the food groups. Previous scores measuring the healthiness and environmental sustainability of the diet use a scoring for the healthiness of the diet (using e.g., a dietary diversity score or nutrient data) and a scoring for the environmental impact (using LCA data) of the diet and combine this in one overall score [18,20]. Another index based on the EAT-Lancet recommendations uses a binary scoring system where the lowest and highest recommended intakes were taken to assess whether participants met each of the recommendations (i.e., above the minimum intake or below the maximum intake) [31]. This index showed inverse associations with NCDs in a UK based population. However, this binary scoring system was shown to be negatively associated with nutrient adequacy in resource poor settings [32]. Hanley-Cook et al. therefore suggest that minimum intake values (>0 g/d) should be used to avoid non-consumers of the nutrient dense dietary components score high on this index [32]. The WISH counteracts this drawback of the binary score by using a continuous scoring on a scale from 0 to 10, along with various sub-scores, rather than a binary scoring system. This allows a gradation in the total score and so gives a more fine-tuned idea on the association with health and the environment and, through the sub-scores, with the particular drivers of the score itself.

For the calculation of the WISH, consumption amounts in grams for the 13 EAT-Lancet food groups are needed. Dietary data obtained from 24 h recalls, (weighed) food records or from a food frequency questionnaire, which includes the relevant food groups and questions on amounts consumed, can be used for this purpose. These dietary intake assessment methods are more intensive to collect and analyse compared to data needed for a basic dietary diversity score, such as Minimum Dietary Diversity-Women (MDD-W), for which only the consumption (not the quantity consumed) of a food group is needed [33].

Some points to keep in mind during further evaluation and adjustments of the WISH related to local applicability, environmental sustainability, healthiness and costs of the diet are discussed below. Generally, the EAT-Lancet recommendations are based on global averages, and therefore not adapted to the local context. Local adaptations, based on cultural dietary practices, could therefore be a necessity. One could, in this case, think of adapting the recommendations for the protein source foods to the local context. Regarding environmental sustainability one could consider sub-groups of the red meat component in ruminant and non-ruminant categories. Emissions from ruminants (from e.g., beef) are generally greater than from non-ruminant species (such as pork) [34] and taking this into account in the WISH scoring could give a better idea of the environmental impact of a diet. Another point to consider when making sub-groups relates to nutrient adequacy or healthiness of the diet, grouping vegetables and fruits in vitamin A rich fruits/vegetables, green leafy vegetables and citrus fruits. Setting recommendations for such sub-groups could increase the association between the WISH score and nutrient adequacy. Though the EAT-Lancet provides recommendations for vegetable sub-groups, it is not clear how the commission came to the recommended intakes and thus this refinement was not included in the WISH score.

Costs of the diet are not considered in the WISH scoring, however, an environmentally sustainable and healthy diet should also be an affordable diet. It is shown that the EAT-Lancet diet is not affordable for poor households [35,36]. Furthermore, it is highlighted that the EAT-Lancet diet is more expensive than a nutrient adequate diet [35]; bringing into question the economic feasibility of adhering to the EAT-Lancet recommendations for a large part of the world’s population.

Evaluation of the feasibility of modeling the environmental impact and healthiness of the diet in one scoring system should be tested with additional datasets. This could lead to refinements in components scoring and food group classifications for health and environmental impact. Evidence on healthy and sustainable diets is growing, different foods, cut-off points for the recommendations or level of impact on the environment for the different food groups for different regions in the world and food, especially production, systems might be desired [20]. Furthermore, associations between nutrient adequacy, disease outcome and other validated sustainable diet quality indices should be part of next steps in the validation process.

## 5. Conclusions

The WISH aims to measure the healthiness and environmental sustainability of the diet with potential application in various global contexts. The recommendations used for the construction of the WISH are the only set developed for global promotion of a planet-friendly and healthy diet. A single globally applicable sustainable diet quality index allows for comparing healthiness and environmental sustainability of the diet across various countries, which is often an important prerequisite of such a metric in food system research. Our initial analysis shows that the WISH is able to differentiate between the healthiness and the environmental sustainability of the diet in Vietnam. Further evaluation of the index should be done across countries using indicators of diet quality and environmental sustainability. Additionally, assessing whether the WISH can reflect changes over time would be an interesting evaluation exercise.

## Figures and Tables

**Table 1:** The components included in the World Index for Sustainability and Health (WISH).

Dietary Component	Healthiness ^1^	Impact on Environment ^2^	Recommended Intake in g/day (Lower and Upper Range of Intake)
Whole grains	Protective	Low	≥125 (100–150) ^3^
Vegetables	Protective	Low	300 (200–600)
Fruits	Protective	Low	200 (100–300)
Dairy foods	Protective	Medium	250 (0–500)
Red meat	Limit	High	14 (0–28)
Fish	Protective	High	28 (0–100)
Eggs	Neutral	Medium	13 (0–25)
Chicken and other poultry	Neutral	Medium	29 (0–58)
Legumes	Protective	Low	75 (0–100)
Nuts	Protective	Medium	50 (0–75)
Unsaturated oils	Protective	Low	40 (20–80)
Saturated oils	Limit	High	11.8 (0–11.8)
Added sugars	Limit	Low	31 (0–31)

^1^ Based on the supplementary material of the EAT Lancet recommendations [22]. ^2^ Based on the assessment of Clark et al. [13] with sustainability indicators: greenhouse gas, land use, eutrophication, acidification, and scarcity weighted water. ^3^ Recommended amount of intake obtained from Global Burden of Disease study [1].

**Table 2:** Components consumption and WISH scoring.

Dietary Component	Non-Consumers (%)	Intakes of Food Groups for All Participants in Gram Mean (SD)	Direction of Change in Intake to Obtain Higher WISH Score
Whole grains	97.5	1.0(6.6)	Increase
Vegetables	0	222.5(100)	Increase
Fruits	46.0	56.7(81.1)	Increase
Dairy foods	96.7	3.3(19.4)	Increase
Red meat	2.8	126.1(90.3)	Decrease
Fish	38.1	35.6(53.2)	Decrease
Eggs	39.6	25.1(29)	Decrease
Chicken and other poultry	55.1	37.7(58.7)	Decrease
Legumes	45.5	61.1(77.3)	Increase
Nuts	84.1	2.3(8.0)	Increase
Unsaturated oils	8.3	6.5(6.1)	Increase
Saturated oils	90.7	0.2(1.1)	Good
Added sugars	81.1	1.1(13.4)	Good

## Data Availability

Restrictions apply to the availability of these data. Data was obtained from Bill and Melinda Gates Foundation (BMGF) and the UK Foreign, Commonwealth & Development Office (FCDO)funded project nr OPP1182727 and are available from Elise F. Talsma with the permission of Wageningen University and Research en Hanoi Medical University.
